# Identifying Opportunities, and Motivation to Enhance Capabilities, Influencing the Development of a Personalized Digital Health Hub Model of Care for Hip Fractures: Mixed Methods Exploratory Study

**DOI:** 10.2196/26886

**Published:** 2021-10-28

**Authors:** Lalit Yadav, Tiffany K Gill, Anita Taylor, Jennifer De Young, Mellick J Chehade

**Affiliations:** 1 NHMRC Centre for Research Excellence in Frailty and Healthy Ageing Adelaide Medical School University of Adelaide Adelaide Australia; 2 Adelaide Medical School, Faculty of Health and Medical Sciences University of Adelaide Adelaide Australia; 3 Department of Orthopaedics & Trauma Royal Adelaide Hospital Adelaide Australia

**Keywords:** digital health, mixed-methods, hip fractures, behavior change, patient education, model of care, mobile phone, patient networked units

## Abstract

**Background:**

Most older people after a hip fracture injury never return to their prefracture status, and some are admitted to residential aged care facilities. Advancement of digital technology has helped in optimizing health care including self-management and telerehabilitation.

**Objective:**

This study aims to understand the perspectives of older patients with hip fracture and their family members and residential aged caregivers on the feasibility of developing a model of care using a personalized digital health hub.

**Methods:**

We conducted a mixed methods study in South Australia involving patients aged 50 years and older, their family members, and residential aged caregivers. Quantitative data analysis included basic demographic characteristics, and access to digital devices was analyzed using descriptive statistics. Spearman rank-order correlation was used to examine correlations between the perceived role of a personalized digital health hub in improving health and the likelihood of subsequent use. Findings from qualitative analysis were interpreted using constructs of capability, opportunity, and motivation to help understand the factors influencing the likelihood of potential personalized digital health hub use.

**Results:**

This study recruited 100 participants—55 patients, 13 family members, and 32 residential aged caregivers. The mean age of the patients was 76.4 (SD 8.4, range 54-88) years, and 60% (33/55) of the patients were female. Approximately 50% (34/68) of the patients and their family members had access to digital devices, despite less than one-third using computers as part of their occupation. Approximately 72% (72/100) of the respondents thought that personalized digital health hub could improve health outcomes in patients. However, a moderate negative correlation existed with increasing age and likelihood of personalized digital health hub use (Spearman ρ=–0.50; *P*<.001), and the perceived role of the personalized digital health hub in improving health had a strong positive correlation with the likelihood of personalized digital health hub use by self (Spearman ρ=0.71; *P*<.001) and by society, including friends and family members (Spearman ρ=0.75; *P*<.001). Most patients (54/55, 98%) believed they had a family member, friend, or caregiver who would be able to help them use a personalized digital health hub. Qualitative analysis explored capability by understanding aspects of existing knowledge, including willingness to advance digital navigation skills. Access could be improved through supporting opportunities, and factors influencing intrinsic motivation were considered crucial for designing a personalized digital health hub–enabled model of care.

**Conclusions:**

This study emphasized the complex relationship between capabilities, motivation, and opportunities for patients, their family members, and formal caregivers as a *patient networked unit*. The next stage of research will continue to involve a cocreation approach followed by iterative processes and understand the factors influencing the development and successful integration of complex digital health care interventions in real-world scenarios.

## Introduction

### Background

The population of South Australia is older than that of all the mainland states and territories in Australia, except Tasmania. According to the Australian Bureau of Statistics, the current population of South Australia is approximately 1.7 million [1]. It is expected to increase to 1.85 million by 2026 and to 2 million by 2038 using current population projections. This increase was reflected by a significant increase in the older population. The number of retirees in the 65-79 years age group is projected to increase by 40% by 2041, using 2016 as the baseline. Moreover, the population aged 80 years and older is projected to increase by 117% over the same period [1], which will require an increased need for appropriate health and social care [2]. Although there has been greater realization that the skills, knowledge, and experience of older people could be better used with regard to their health care, there remains a view that they are a drain on society given their health problems and service needs [3,4]. The United Nations Economic Commission for Europe has suggested that altering this view is a key strategy to improve the integration and participation of older people in society [5].

### Hip Fractures and Multimorbidity

Fragility fractures mostly occur in older people owing to low-trauma falls, which often result from multimorbidity [6,7]. Multimorbidity is the presence of more than one chronic disease in an individual and is influenced not only by health-related characteristics but also by socioeconomic, cultural, and environmental factors, as well as patient behavior [8]. Hip fractures are among the most devastating fragility fractures, and their management becomes challenging because of the required involvement of several disciplines within health and social care. This cohort not only represents healthy older people at one end of the spectrum but also comprises people with frailty, sarcopenia, osteoporosis, and dementia at the other end of the spectrum. This makes management of an acute event such as a hip fracture complex, with wide-ranging outcomes within the health care systems involving multiple disciplines and service providers [9-11]. It is made even more complex with the crossover between different levels of care, ranging from acute tertiary to primary and residential aged care [12,13]. Most patients who are admitted to acute hospital care are unable to return to their prefracture level of independence [14-16]. Although some patients return to independent living in their own homes, a significant number are either newly admitted or return to residential aged care [17]. Thus, we believe that individual patient outcomes can only be improved by envisaging a model of care that ensures a holistic and integrated approach to health service delivery while empowering patients and their caregivers.

### Digital Health–Enabled Models of Care

Models of care (MoCs) are frameworks mutually agreed by key stakeholders accountable for delivering evidence-informed quality health care. Such frameworks must be functional, outlining the optimal manner in which condition-specific care should be made available and delivered to consumers while addressing issues related to specific aspects of service provision [18]. They go beyond clinical practice guidelines to incorporate practical delivery issues of who, when, where, and how care is best delivered and evaluated [18]. Thus, MoCs become complex due to their multidisciplinary workforce links to secondary and tertiary care services, the biopsychosocial needs of the patients, and frequently changing organizational structures. Although the mandate of primary care is to offer a generalist approach for dealing with older adults with multimorbidity, the coordination of community services is difficult. It is often left to the patients and their caregivers to coordinate and navigate through a range of services into which their individual social circumstances and priorities also need to be factored [18]. Provision of accurate, timely, and adequate information by educating patients plays a vital role in improving engagement and participation in the recovery and rehabilitation processes within the MoC. Health professionals often overlook patients’ health literacy during routine practice, incorrectly assuming that the health information and instructions provided to patients and their family members have been understood [19,20]. Patient education, which also involves family members and residential aged care staff, is crucial for empowerment and improving health literacy [7,21-23].

Technological advancements have led to the evolution of clinical decision support systems and a myriad of consumer mobile apps to target different stakeholders, with the intention of optimizing health care and self-management of chronic disease conditions and maintaining a healthy lifestyle [12,13,24]. Nevertheless, there remains a need to build on the knowledge exchange process between health care providers and patients, along with their family members and caregivers, acting as facilitators [13]. By targeting different multimorbidities, which correspond to the internal capacity of individual older people, care can be personalized. This aligns with the World Health Organization (WHO) guidelines on community-level interventions to manage declines in intrinsic capacity through an integrated care approach for older people (WHO-Integrated Care for Older People) [13,23]. The WHO describes digital health as a broad umbrella term encompassing eHealth, mobile health, and emerging areas, such as the use of advanced computing sciences in big data, genomics, artificial intelligence, and machine learning [25]. Commitment and strategic engagement of stakeholders, including patients and the community, is required to improve health care services across all stages, from inception to operation or implementation [25]. Further advancement of these technological solutions can bypass some of the care disparities imposed by sociodemographic and geographic barriers and support the move toward universal health coverage [26,27].

### Objective

The aim of this study is to understand the perspectives of older patients with hip fracture, their family members, and formal caregivers in residential aged care facilities to inform the development of a personalized digital health hub by understanding their current access to digital devices and factors affecting the likelihood of future use [8].

## Methods

### Setting and Study Design

We examined patients with hip fracture aged 50 years and older. This mixed-methods study [28] was conducted at the Royal Adelaide Hospital, a tertiary trauma care center in Adelaide, South Australia. This is one of the busiest hospitals in Australia for acute hip fractures, with local estimates suggesting approximately 500 to 600 patients treated annually [29].

### Digital Health Hub Initial Concept

In this study, a digital health hub scenario, which is currently under development, was described to generate appropriate responses from the study participants. This proposed web-based health information portal, or a website, is intended for patients who can access all relevant information about their hip fractures. It includes details in multimedia formats of diagnosis and treatment options, medications, wound management and rehabilitation exercises, potential problems encountered during the hospital admission and post discharge, information on how to deal with difficulties, as well as how and when to attend follow-up appointments or seek more help from the health care team. It is interactive, enabling patients and their caregivers to provide both targeted and patient-initiated information to their health care provider, which is captured digitally. It also allows users to selectively make information available to family members or other people involved in their care (either formally or informally).

### Data Collection and Analysis

Participants in this study were recruited from a previous prospective cohort study that focused on the delivery of fracture liaison service, undertaken between January and December 2016. Patients were contacted consecutively, and those who consented were invited to participate in the study. If participants in the original study had caregivers participating on their behalf, they were approached in a similar manner. Family members were represented as informal caregivers, whereas formal caregivers were caregivers of older people in residential aged care facilities. The data for this study were collected over a period of 6 months, from January 2017 to June 2017, using face-to-face interactions or telephone calls on the basis of individual preferences. A semistructured questionnaire consisting of closed and open-ended questions was developed and administered (Multimedia Appendix 1). Participants’ responses to each question were entered into a hard copy Word (Microsoft Corporation) document by the research staff while administering the survey questionnaire. These responses were then compiled on an Excel (Microsoft Corporation) spreadsheet and stored on a password-protected folder on the secured server of SA Health.

Quantitative data analysis included basic demographic characteristics, and access to computers and the internet (digital devices) at home and in the workplace were analyzed using descriptive statistics. Spearman rank-order correlation was used to examine correlations between the perceived role of a personalized digital health hub in improving health and the likelihood of subsequent use of a personalized digital health hub. Fisher exact test and odds ratios were calculated for comparisons across respondent groups with respect to previous access to computers at the workplace, gender differences, and likelihood of potential personalized digital health hub use.

**Figure 1:**
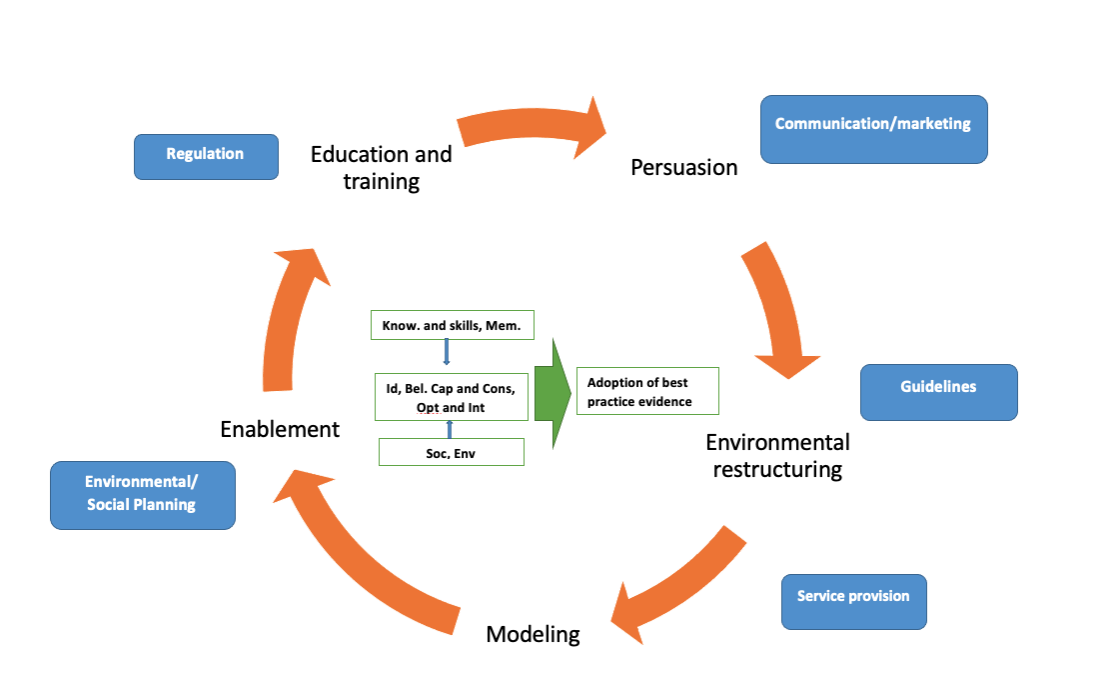
Constructs of capability, opportunity, and motivation embedded within a Behaviour Change Wheel Framework [27]. Sources of Behaviour box and arrow in green; Intervention functions orange; Policy categories blue. Know: knowledge; Mem.: memory, attention and decision processes (capability); Id: social or professional role and identity; Bel. Cap. and Cons: beliefs about capabilities and consequences; Opt. and Int.: Optimism and intentions (motivation); Soc.: social influences; Env.: environmental context and resources (opportunity).

Qualitative data analysis included a series of open-ended questions to identify potential barriers and facilitators for accessing personalized digital health hub. Responses to the open-ended questions were analyzed deductively and aligned with the tenets of capability, opportunity, and motivation [30,31]. These findings interpreted using constructs of capability, opportunity, and motivation embedded within a theoretical Behavior Change Wheel (BCW) framework and helped to understand factors influencing the likelihood of potential personalized digital health hub use. The use of this framework to explore multidisciplinary stakeholder engagement within hip fracture management has been described elsewhere (Figure 1) [31,32].

### Ethics

This study was approved by the Human Research Ethics Committee of the Central Adelaide Local Health Network (RAH protocol number R20080704, HREC reference: 080704, ethics approval amendment on 12/12/2016, CALHN reference number: 8977, SSA approval 23/1/2017).

## Results

### Quantitative Findings

Overall, 100 participants were recruited in the study (Table 1). These included 55 patients, 13 family members as informal caregivers, and 32 residential aged care workers. The age (in years) of patients (mean 76.4, SD 8.4; range 54-88) was similar to that of their family members (mean 77.2, SD 10.0), whereas the residential aged caregivers were younger (mean 45.2, SD 11.6). Females represented 60% (33/55), 54% (7/13), and 86% (28/32) of patients, family members or informal caregivers, and residential aged formal caregivers, respectively. Around a quarter to a third of the participants were from *professional* occupations across all 3 groups. The residential care group included 5 registered nurses and 2 enrolled nurses. Within the patients’ group, the common occupations were laborers, clerical and administrative workers, homemakers, machinery operators, and drivers.

With respect to digital access, approximately half of the patients (28/55, 51%) and their families (6/13, 46%) had access to their own computer devices through the internet. A quarter (13/55, 24%) of the patients used a computer as part of their occupation in comparison to 31% (4/13) of family members, whereas more than 91% (29/32) of the residential aged caregivers had computer access.

Patients who reported using a computer as part of their work were 8 times more likely to have access to a computer with internet access at home compared with those who did not use a computer at work (odds ratio [OR] 8.08, 95% CI 1.58-41.18; Fisher exact test=0.0095; *P*=.05). The mean age of those with access to a computer was 4.6 years less than that of those without access (74.2 vs 78.8, *P*=.04).

Approximately 40% (25/68) of the patients and their family members reported using basic operational tools such as email and Google (or other search engines) in comparison to 100% (32/32) of the residential caregivers who used these functions (Table 1). Of these, more than 85% (46/53) of the patients found it *reasonably easy* to *very easy* to operate these basic functionalities through the internet. Skype or other video calling programs were used by only a quarter of patients and their family members, whereas 44% (14/32) of the residential caregivers reported that they used these programs. Among the patient group, men were 3 times more likely to report having used email than women (OR 3.75, 95% CI 1.17-11.9; *P*=.02). However, given the opportunity, 42% (23/55), 38% (5/13), and 56% (18/32) of the patients, their family members, and residential aged caregivers, respectively, expressed their willingness to learn or advance their skills in these areas. While exploring this aspect further, all patients except 1 (54/55, 98%) also said they had a family member, friend, or caregiver who would be able to help them use a digital health platform.

Approximately 72% (72/100) of the respondents thought that personalized digital health hub could improve the health of patients. Although a moderate negative correlation existed with increasing age and likelihood of personalized digital health hub use (Spearman ρ=–0.50; *P*<.001), the perceived role of the digital health hub in improving health had a strong positive correlation with the likelihood of personalized digital health hub use by self (Spearman ρ=0.71; *P*<.001) and by society, including friends and family members (Spearman ρ=0.75; *P*<.001). Furthermore, those participants who thought that the support content and services provided through personalized digital health hub would improve their health were more likely to use such a platform by themselves (OR 33.80, 95% CI 7.33-155.76; *P*<.001), and their friends and family members (OR 27.23, 95% CI 8.06-91.95; *P*<.001).

In terms of intention to buy a computer, 65% (36/55) of the patients said they would not be willing to purchase a computer or other device to enable them to access a web-based portal. Of the 35% (19/55) who would be willing to purchase a computer or device, 13% (7/55) said they would be willing to spend up to Aus $200 (US $144), 18% (10/55) said they would spend up to Aus $500 (US $360), and 4% (2/55) said they would spend up to Aus $1000 (US $720).

**Table 1:** Basic demographics and computer access characteristics.

Demographics and access to digital technology			Patients (n=55)		Family members (n=13)		Residential aged caregivers (n=32)
Age (years), mean (SD)			76.4 (8.4)		77.2 (10.0)		45.2 (11.6)
Female, n (%)			33 (60)		7 (54)		28 (86)
**Occupation^a^, n (%)**							
	Managers	2 (4)		1 (8)		N/A^b^	
	Professionals	13 (24)		4 (30)		7 (22)	
	Technicians and trade workers	6 (11)		2 (14)		N/A	
	Community and personal service workers	1 (2)		N/A		25 (78)	
	Clerical and administrative workers	7 (13)		1 (8)		N/A	
	Sales workers	3 (5)		1 (8)		N/A	
	Machinery operators and drivers	5 (9)		1 (8)		N/A	
	Laborers	8 (14)		1 (8)		N/A	
	Homemaker	5 (9)		1 (8)		N/A	
	Unemployed	1 (2)		N/A		N/A	
	Did not respond	4 (7)		1 (8)		N/A	
**Access to digital technology**							
	Use of computer	13 (24)		4 (31)		29 (91)	
	Own computer with internet access	28 (51)		6 (46)		32 (100)	
	Device access but no internet	4 (7)		0 (0)		0 (0)	
	Use email	20 (36)^c^		5 (38)		32 (100)	
	Use Google or other search engines	20 (36)		5 (38)		32 (100)	
	Use Skype or other video calling programs	13 (24)		3 (23)		14 (44)	
	Willingness to learn^d^	23 (42)		5 (38)		18 (56)	

^a^Occupation groups as defined by the Australian and New Zealand Standard Classification of Occupations.

^b^N/A: not applicable.

^c^Men versus women, odds ratio 3.75 (95% CI 1.17-11.9; *P*=.02).

^d^Willingness to learn how to use email or internet search engines such as Google or a video calling program such as Skype, if the respondents have not used any of them before.

### Qualitative Findings

The respondents answered the two open-ended questions within the survey instrument to explore barriers (Q15) and facilitators (Q16) influencing the likelihood of using a personalized digital health hub to educate and empower patients, their family members, and caregivers within residential aged care (Multimedia Appendix 1). These factors were interpreted using constructs of capability, opportunity, and motivation within a BCW framework, and relevant quotes from the study respondents (R) were also provided. Capability in this study was defined as an individual’s psychological and physical capacity to engage with the potential personalized digital health hub, which included having the necessary knowledge and skills. Opportunity considered all the factors lying outside the individual that make the behavior possible or prompt it, such as the likelihood of engaging with the potential personalized digital health hub. Motivation included processes that energize and direct behavior, not just goals but also habitual processes, emotional responses, and analytical decision-making. These constructs influence each other, as they work dynamically, such as access to opportunity can drive motivation, whereas enacting behavior can alter capability, motivation, and opportunity [32]. There were 59 participants who responded to questions corresponding to barriers with further breakdown of 38, 9, and 12 as patients, their family members, and residential aged caregivers, respectively. In contrast, 40 participants responded to questions corresponding to facilitators, representing 24, 3, and 13 patients, their family members, and residential aged caregivers, respectively.

### Capability

Some patients recognized that possessing the necessary knowledge and skills while accessing digital devices can help explore relevant web-based health information, which could enable a better understanding of their health condition. Conversely, there were some family members and caregivers who lacked confidence in using digital devices. There was no one available to teach them and also felt inadequate about understanding the patient’s medical condition. The patients’ comments reflect that the information gathered through such a digital health platform would actually help improve their decision-making during the recovery process:

Can't use the computer, no one to teach me to use it at the moment.

R53

Would have been very useful (internet), always looking things up anyway.

R49

Keeping up with computers and technology keeps me sharp [informing decisions].

R38

### Opportunity

Patients and their family members considered their personal environment and the affordability of resources, such as digital devices, as a major limiting factor. Residential aged caregivers saw digital health platforms as an opportunity to provide general health information, including healthy lifestyle, diet, and exercise. Furthermore, it was suggested that a platform such as a personalized digital health hub (or similar) would be more efficient or easier than existing options and would provide a potential solution with resources consisting of videos of exercises such as yoga and tai chi. In addition, several patients were of the opinion that a list of available services (eg, allied health professionals, exercise classes, alternative therapies) along with health management information could be well received by the community:

Can't afford computer on aged pension.

R15

Lots of people interested in general information about their health as well ie healthy lifestyle, diet, exercise.

R72

Videos [exercises] would be very helpful, would like information about how long hip replacements last and how to take care of them, any information is good information? Could include tai chi or yoga.

R24

Knowing it’s there [digital platform] to fall back on, list of things/services that are available.

R47

Rehab exercises, suggestions and options, a list of services.

R67

### Motivation

Being older was identified by both patients and their family members as one of the main hurdles to using the potential personalized digital health hub platform. Residential caregivers, however, identified practical issues such as lack of time in their existing role to use such a solution, which is currently not a part of their job. Participants across all 3 categories identified their existing capabilities as a limiting factor. However, they were also positive about the potential capabilities of a digital solution, such as the availability of information that would reduce the need to visit a physician and access to trustworthy interventions. These interventions include videos and information about health and instructions from reputable sources such as physiotherapists that they can follow in their own time as well as the potential to more easily track their appointments with different health care providers:

Hard for other elderly people.

R8

Just not practical, responsible for more than one person at a time so time using this would take away from actually caring for people.

R83

Access to information on demand, not have to visit doctor, not missing phone calls and use on own time.

R3

Reputable sources would be good, videos of exercises helpful, a realistic timeline for recovery would be useful.

R43

The residential aged care staff thought that a digital health solution could potentially improve handover processes through a better exchange of information between specialists and caregivers. Most participants were optimistic about the range of functions that a digital health platform could provide; however, some had reservations such as preferring phone conversations or maintaining conventional face-to-face interactions with the physician. Emotionally, some consumers were unhappy with the services provided through technology-based solutions in comparison with face-to-face interactions. One of the patients identified a potential lack of reinforcement in terms of someone who could teach or handhold, which could be a barrier to using a digital solution. Conversely, some patients thought that it could help them achieve more peace of mind and service satisfaction:

Would (digital platform) improve handover of information between specialists and carers or the patient, keep everyone on the same page more.

R88

Prefer phone calls, more personal, know who you're talking to.

R14

Good to be able to see exercises [over the internet], peace of mind.

R6

## Discussion

### Principal Findings

This study was considered as one of its kind due to the involvement of combined perspectives from patients with hip fragility fractures, their family members, and residential aged caregivers. Older people with hip fractures often have low intrinsic capacity, leading to depletion in physical function, mental health impairment, and increased health care costs [33]. Therefore, it is crucial to address hip fractures among older people, particularly those with multimorbidities, as a whole and in an integrated manner, rather than managing individual issues in isolation or silos, including improving health literacy by connecting with family members and formal caregivers as networked units [34]. This study explored the feasibility of a potential personalized digital health hub model of care in educating, empowering, and integrating health services, including self-management, for older patients with hip fractures in South Australia. Quantitative and qualitative methods were used in synergy to maximize the interpretation of findings. The BCW framework was applied through constructs of capability, opportunity, and motivation. These constructs are embedded within the BCW framework, which has been used in many contemporary scenarios for developing complex health interventions, including stroke rehabilitation [35] and multiple lifestyle issues [36,37].

Quantitative findings suggested that patients and their family members were of the same age, and almost half of them had current access to digital devices with the internet, despite only about a quarter of them using computers as part of their occupation (Opportunity). Although significant gaps existed with respect to operating emails, video calling, and exploring search engines (Capability), many of them expressed their willingness (motivation) to advance their skills through the supporting environment. The latter can be strengthened as 98% (54/55) of the patients said they had family members, friends, or caregivers (Opportunity) who would be able to help them use such a resource. Furthermore, the findings from our study suggest that 72% (72/100) of the respondents thought that the personalized digital health hub would be useful for improving their health.

Findings from the qualitative analysis explored deeper meanings of individual capability, opportunity, personal circumstances, and motivational factors varying within each group. Capability mainly focuses on knowledge, skills, and decision-making processes, whereas opportunities could be in the form of availability and access to digital devices and holistic care [10], including healthy lifestyle, diet, and exercise [36]. Some consumers may have better knowledge and skills to understand health information and access web-based resources. These people advocated for the personalized digital health hub being available for communicating high-quality and trustworthy health information resources, tracking appointments, and linking relevant services through a single hub. On the other hand, some preferred traditional face-to-face interactions and considered declining individual capacities due to aging as a possible challenge to cope with learning associated with the new technology. For some, personal circumstances, including affordability and access to computer systems, were important aspects to be considered. Furthermore, the findings suggested that motivation to engage with personalized digital health hub could be adversely affected by increasing patient age and contributing to additional workload for caregivers. However, information available in different formats, such as video or interactive, could improve patient engagement, help navigate different service provisions, and improve workforce handover processes delivered through an agreed model of care [18].

The sharing of information between patients and health care professionals is one of the key pillars of therapeutic relationships [38]. Increasingly, this information is being shared on the web, as digital health platforms through which patients can access education regarding medical conditions, information on self-management, and communication of health information to health professionals [39-41]. Clearly accessing and using some of these platforms will challenge certain groups within populations, such as the older adults. Ulrich and Vaccaro [42] described the benefits to patients receiving health information on the internet. These included improved health outcomes, mainly due to fulfillment of expectations and changes in behavior, which are facilitated by improved availability of information and resources. They note that older people in particular prefer audiovisual or pictorial explanations and information [42]. Furthermore, most patients do not have the capacity to distinguish nonbiased and reputable sources of information from commercially biased promotional materials [42]. The personalized digital health hub research program described here presents an opportunity to standardize and ensure the quality and evidence base of information received by patients and caregivers. A contemporary example of a digital technology used to improve access to first-line care for musculoskeletal conditions is the painHEALTH initiative [43]. This platform was codeveloped with consumers in response to the escalating burden of pain management associated with musculoskeletal conditions. The development of content was aligned to best practice recommendations from musculoskeletal MoC [18,43] and calls to action for improved care highlighted in the Australian National Pain Strategy [44].

Not all people have access to the internet, and this is especially true for people older than 65 years. However, according to the Australian Bureau of Statistics and Household Use of Information Technology survey for 2016-2017, the proportion of users accessing the internet for health-related services or research has more than doubled from 22% in 2014-2015 to 46% in 2016-17. Among older people, 55% of those aged 65 or older accessed the internet in a typical week, a 4% rise from the survey conducted in 2014-2015 [45]. Internet use correlated positively with educational attainment and household income and negatively with rurality. However, the survey is likely to be an overestimate of the proportion of older adults in the population who regularly use the internet as it excluded *people living in nonprivate dwellings such as hotels*, *university residences*, *students at boarding schools*, *patients in hospitals*, *inmates of prisons*, and *residents of other institutions* (eg, *retirement homes* and *homes for persons with disabilities*) [45]. The survey also noted that 14% of Australian households did not have internet access [45].

Caregivers, spouses, or family members can and should be engaged to assist with the personalized digital health hub platform in consultation with the patient. However, any provision to replace in-person clinical interactions must include a safety net for patients without access. Our study suggests that 46% (46/100) of the participants were willing to learn and develop their skills. Usually, patients accessing public tertiary care facilities are more likely to belong to the lowest socioeconomic status grouping [46]; despite being economically disadvantaged, more than one-third of these patients within our study were willing to buy a computer or other smartphone devices to access the potential personalized digital health hub platform. The majority of them would spend between Aus $200-$500 (US $144-$360), but very few of them could go up to Aus $1000 (US $720). This emerging likelihood of using potential digital health solutions was also supported by another study [47], in which 63% of the participants expressed their intentions as *definitely or probably* to be using a digital health platform as a web-based interface. Such a platform could carry out tasks such as making appointments, asking questions, receiving treatment, information, and providing support for their health and well-being. This study also emphasized the importance of user-friendliness and quickly resolving issues such as bugs in the initial releases [47].

Although digital health care may offer feasible and efficient options for monitoring and securely interacting with patients, an adequate level of engagement with the technology by all stakeholders is critical. In a recent rollout of an Australian opt-out digital health data management system (known as My Health Record), although there was a 90% subscription rate by patients, less than a quarter of health provider organizations were using the system [48]. This was even though 60% ranked clinical integration and improved patient experiences as their top priorities [49]. This mismatch suggests that educating practitioners to use digital systems is as important as patient engagement and compliance [50,51]. Together, this could have an incremental effect on patient outcomes and service delivery.

### Limitations

This study has several limitations. One of them was a convenience sample from a single hospital site. However, this site is a major tertiary referral center that receives hip fracture patients across South Australia. Similarly, because of time and resource constraints, we were only able to recruit 100 participants in this study. We recommend that future studies consider a large sample size and further build on the evidence [52,53]. Another limitation is that patients with impaired cognition and high multimorbidity risk were not included. However, we attempted to engage residential aged caregivers who received many of these patients. These caregivers certainly act as facilitators, helping their patients, and are equally important stakeholders in the care process. Our study highlighted, from the perspective of caregivers, that personalized digital health hub could improve the handover process between specialist care and residential age care. [13]. We also acknowledge that these data were collected in 2017; however, we consider the findings from the study to be unique and still relevant, on the basis of the existing literature and development in the area of broader musculoskeletal care and digital health. Nonetheless, studies demonstrate the need to substitute current inefficiencies of siloed health care models with more person-centered and integrated models in which the patients and their caregivers are empowered as a team that works toward a personalized health solution to illness [8,18,51]. We acknowledge that when this study was conducted, the concept of personalized digital health hub was very theoretical; however, with inputs from other study components, we have been able to advance it to the stage of a prototype to be tested in the practice setting in the next stage of our research activity. Another limitation offered by the design of this study was a weak component of qualitative methodology, as the primary data collection tool consisted of only 2 open-ended questions. However, despite this limitation, we attempted to maximize the relevance of findings by applying the analytical behavior change framework. Similarly, in this process, the increased use of digital technologies to support health care is inevitable, particularly in the context of COVID-19, which has not only accelerated the willingness of health care practitioners to adopt telehealth options but also resulted in patients quickly adapting to and embracing these recent changes [54-56].

### Conclusions

Recovery from fragility fractures among older people requires input from multiple specialties within medicine and allied health domains depending on the presence of concurrent medical conditions. Rather than approaching patients as isolated individuals, we need to consider them in the context of a network of caregivers and delivery of service as an integrated holistic model of care. Findings from this study contributed to understanding the capabilities, motivation and opportunities of patients, family members and formal caregivers as a *patient networked unit* rather than as siloed groups and provided a proof of concept around a personalized digital health hub [8]. This will provide greater cohesion and opportunities for success while navigating through a complex recovery pathway with multiple caregivers and is critical to the development of a personalized digital health hub–enabled MoC. Future paths will also incorporate perspectives from other relevant stakeholders as part of the *patient networked unit*, evolving through iterative processes and cocreation, to improve our understanding around the successful development of complex health care interventions and its drivers [13,57].

## Appendix

Multimedia Appendix 1 In-depth interview schedule (stakeholders).

## Abbreviations

BCW: Behavior Change Wheel
MoC: model of care
OR: odds ratio
WHO: World Health Organization
