# Supplementary material for: Identifying Opportunities, and Motivation to Enhance Capabilities, Influencing the Development of a Personalized Digital Health Hub Model of Care for Hip Fractures: Mixed Methods Exploratory Study
Source: J Med Internet Res. 2021 Oct 28;23(10):e26886. doi: 10.2196/26886 (PMC8587193; doi:10.2196/26886)
Supplement: Multimedia Appendix 1 [file jmir_v23i10e26886_app1.docx]

**Multimedia appendix 1: In-depth interview schedule (Stakeholders)**

Thank you for consenting to be part of this research. Through this interview, I wish to explore your perspective around potential development of an electronic health information education platform for older people with a hip fracture injury.

Name:_____________________________Age____/years Sex______

Designation____________________Department/Hospital_____________________

Date of consent_________________________

**Context**

Question-1: What is your experience, in general, with older people with hip fractures admitted to the hospital or attending clinic/consultation for treatment?

*Probe: physical status, gender, education level, empowerment, priority, recovery needs, care pathway, role of carer*

Question-2: What is your perspective, particularly around health literacy in this group of population?

*Probe: health information needs/areas, existing provision, difficulty in accessing, possible improvement solutions, ehealth*

**Content**

Question-3: In your opinion, what are the important areas of health information for patients recovering from a hip fracture injury after their discharge from the hospital?

*Probe: clinical recovery, functional improvement, WHO ICOPE, multiple medical conditions, other issues*

Questiona-4: In your opinion, do you think there is a need or scope for improving the quality of health information for this group of patients? Do you have any possible suggestions or solutions in this direction?

*Probe: barriers and facilitators, role of carers and community providers (health and social care), different platforms including ehealth or IT solution*

**System**

Question-5: Can you please recollect from your previous experience of coming across any electronic platform used for patient education and/or service delivery? Or any comment in general about the use of IT/ehealth solutions?

*Probe: setting, usage and function, challenges, possible learning and suggestive improvements*

Question-6: Can you please elaborate factors, in your opinion, needs to be taken into consideration while designing an ehealth platform for such group of patients?

*Probe: system functionalities, patient response, existing system integration*
